# Parent–offspring conflicts, “optimal bad motherhood” and the “mother knows best” principles in insect herbivores colonizing novel host plants

**DOI:** 10.1002/ece3.267

**Published:** 2012-07

**Authors:** Carlos García-Robledo, Carol C Horvitz

**Affiliations:** Department of Biology, University of MiamiP.O. Box 249118, Coral Gables, Florida 33124-0421

**Keywords:** Exotic plants, feeding preference, larval and adult survival, oviposition preference, parent and offspring performance

## Abstract

Specialization of insect herbivores to one or a few host plants stimulated the development of two hypotheses on how natural selection should shape oviposition preferences: The “mother knows best” principle suggests that females prefer to oviposit on hosts that increase offspring survival. The “optimal bad motherhood” principle predicts that females prefer to oviposit on hosts that increase their own longevity. In insects colonizing novel host plants, current theory predicts that initial preferences of insect herbivores should be maladaptive, leading to ecological traps. Ecological trap theory does not take into account the fact that insect lineages frequently switch hosts at both ecological and evolutionary time scales. Therefore, the behavior of insect herbivores facing novel hosts is also shaped by natural selection. Using a study system in which four *Cephaloleia* beetles are currently expanding their diets from native to exotic plants in the order Zingiberales, we determined if initial oviposition preferences are conservative, maladaptive, or follow the patterns predicted by the “mother knows best” or the “optimal bad motherhood” principles. Interactions with novel hosts generated parent–offspring conflicts. Larval survival was higher on native hosts. However, adult generally lived longer on novel hosts. In *Cephaloleia* beetles, oviposition preferences are usually associated with hosts that increase larval survival, female fecundity, and population growth. In most cases, *Cephaloleia* oviposition preferences follow the expectations of the “mothers knows best” principle.

## Introduction

The life cycle of most insect herbivores is closely associated with one or a few host plants (Futuyma and Moreno 1988). This ubiquitous specialization stimulated the development of several hypotheses on how natural selection should shape feeding and oviposition preferences of insect herbivores (Mayhew 2001; Craig and Itami 2008). For example, larval stages usually have limited mobility, feeding on the host plant selected by their mothers (Craig et al. 1986; Scheirs et al. 2000; Agosta and Klemens 2009; García-Robledo et al. 2010). It is expected that natural selection will favor females that are able to discriminate among potential hosts and oviposit on plants that will increase offspring's survival (Craig and Itami 2008). This predicted maximization of fitness through offspring's survival is known as the “naïve adaptionist” or the “mother knows best” hypothesis (Scheirs et al. 2000; Mayhew 2001). This hypothesis is the main assumption of most models describing the evolution of host plant choice (Bernays and Graham 1988).

When larvae and adults feed on the leaf tissue of more than one host plant, it is possible that hosts that maximize offspring's growth and survival are not the same host plants that increase adult longevity. This situation can generate parent–offspring conflicts (Scheirs et al. 2000). In such cases, female fitness can be maximized in two different ways. Females may prefer to oviposit on the host plants that increase offspring survival, as predicted by the “mother knows best” principle (Mayhew 2001). An alternative is that adults will spend more time feeding and laying eggs on the host plant that increases their own longevity, even if the consequence of this behavior is a reduction of offspring survival. Such scenario is known as the “optimal bad motherhood” principle (Mayhew 2001).

The “mother knows best” and “optimal bad motherhood” principles represent two contrasting processes involved in the evolution of diets of insect herbivores. “Mother knows best” strategies will promote specialization and diet conservatism, as oviposition preferences will maintain already established plant–herbivore interactions (Scheirs et al. 2000). “Optimal bad motherhood” strategies will promote diet generalization and host shifts as larval stages will need to adapt to less suitable host plants (Scheirs et al. 2000).

Current preference–performance theories of host use focus on well-established plant–herbivore interactions that have persisted enough time for natural selection to have acted (Scheirs et al. 2000). However, plant–herbivore associations are extremely dynamic (Futuyma 1996). Over ecological and evolutionary time scales, both plant and insect herbivores may change their geographic ranges generating novel plant–herbivore interactions (Futuyma 1996). For insect herbivore species to persist, natural selection should promote feeding and oviposition behaviors that increase fitness through local adaptation, but also facilitate diet expansions to novel hosts. Recent evidence shows that traits associated with the ability of colonizing novel hosts are not lost during insect herbivores specialization and the ubiquitous specialization of insect herbivores does not lead to evolutionary dead ends and insect species extinctions (Janz et al. 2006; Garcia-Robledo and Horvitz 2011).

Understanding patterns of preference–performance and the colonization strategies of insect herbivores confronting novel host plants is of major ecological and evolutionary significance (Garcia-Robledo and Horvitz 2011). Colonization of novel hosts represents an important source of ecological and evolutionary novelty, the first step for processes of local adaptation and speciation.

Understanding the behavioral and physiological responses of insect herbivores confronting novel hosts is also an important conservation issue. During the last five centuries, human-driven introduction of exotic species are generating a global reconfiguration of plant and insect communities (Chapin et al. 2000). The effects of these novel plant–herbivore associations on the survival of native herbivore populations will depend on the initial feeding and oviposition behaviors of native herbivores when confronting novel hosts (Camara 1997; Verhoeven et al. 2009).

Insect survival is usually reduced during early colonization of novel hosts (Gripenberg et al. 2010; Harvey et al. 2010). This high mortality on novel hosts is assumed to be the result of local adaptation of herbivores to their native host plants (Futuyma and Moreno 1988). When insect herbivores get into contact with novel hosts, a potential outcome is that locally adapted larvae and adults will show higher preference and performance for their native than for the novel host plants (Harvey et al. 2010).

Another potential outcome is that the initial preferences of insect herbivores for the novel host are maladaptive (Keeler and Chew 2008). Novel hosts may elicit conflicting behavioral and physiological responses by the herbivore. For example, cuticular plant chemicals could elicit strong feeding and/or oviposition preferences for the novel host even in cases when it reduces herbivore fitness (Schlaepfer et al. 2002; Keeler and Chew 2008). These choices that make little adaptive sense are described as ecological traps (Schlaepfer et al. 2002). Herbivores will be able to escape such ecological traps if the insect population displays enough genetic variance to evolve avoidance or higher survival on the novel host (Schlaepfer et al. 2002).

In holometabolous insect herbivores, growth, reproduction, and dispersal functions are segregated among insect's life stages (Pierce and Berry 2011). Larvae specialize in feeding and growing. Adults are the reproductive stage that plays a central role in dispersal and colonization of suitable habitats (Pierce and Berry 2011). Larval stages usually have higher energetic requirements than adults, as they must overcome the challenges of growing fast, complete metamorphosis, and in some cases accumulate resources that will determine adult fecundity (Pierce and Berry 2011). One consequence of these contrasting lifestyles is the potential generation of parent–offspring conflicts during the colonization of novel hosts, as larval stages might be more sensitive to changes in diet than adults (Trivers 1974).

Parent–offspring conflicts can reach extremes where novel hosts can simultaneously reduce larval survival and increase adult longevity. Such situations, described at least for one herbivore, the leafmining fly *Chromatomyia nigra,* is of particular interest to understand the processes involved in colonization and adaptation to novel hosts (Scheirs et al. 2000). One possibility is that oviposition preferences are conservative and females will select the host plant that increases offspring's survival, as predicted by the “mother knows best” principles. An alternative is that feeding preferences, female longevity, and oviposition preferences are positively correlated. In this case, females will oviposit on plants that increase adult longevity but reduce offspring survival, as predicted by the “optimal bad motherhood” principle (Scheirs et al. 2000).

One of the oldest and most conservative plant–herbivore interactions is the association between beetles from the genus *Cephaloleia* and plants from the order Zingiberales. *Cephaloleia* beetles radiated in the Neotropics, feeding on Zingiberales for the last 40–60 million years (Wilf et al. 2000; García-Robledo and Staines 2008). *Cephaloleia* beetles are also known as the rolled-leaf beetles because adults feed and mate inside the scrolls formed by the young leaves of their host plants (García-Robledo et al. 2010). At La Selva Biological Station, a tropical rain forest in Costa Rica, Central America, five exotic Zingiberales from South America, and the Paleotropics are currently colonizing primary and secondary forests (García-Robledo 2010). At present, both generalist and specialist species of *Cephaloleia* beetles are expanding their diets to exotic Zingiberales (García-Robledo and Horvitz 2011).

Using *Cephaloleia* beetles currently expanding their diets to exotic Zingiberales as our study system, the aim of this study was to explore the initial relationships between feeding choices, insect survival, and oviposition preferences during the initial colonization of novel hosts. We were particularly interested in determining if: (1) diet expansions to novel hosts represent ecological traps, and the initial feeding and oviposition choices are maladaptive, reducing both larval and adult survival; (2) feeding and oviposition preferences are conservative, and insect herbivores initially prefer to feed and oviposit on native hosts; or (3) if larval and adult stages diverge in their preferences and performance when feeding on native and novel hosts, generating parent–offspring conflicts.

In cases when diet expansions to novel hosts resulted in parent–offspring conflicts, we explored the relationships among larval and adult survival and oviposition preferences to determine if: (1) females prefer to oviposit on the host plants that maximize their offspring survival, as proposed by the “mother knows best” principle, or (2) if females prefer to oviposit on the hosts that increase adult longevity regardless of an increase in offspring mortality, as proposed by the “optimal bad motherhood” principle.

## Methods

### Study site and species

**Figure 1:**
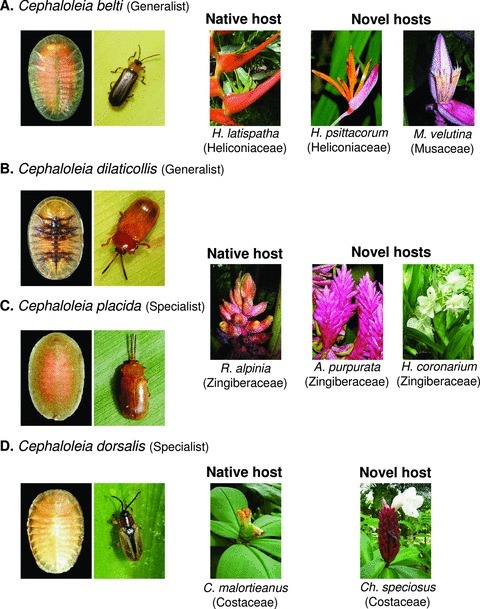
Larvae and adults of *Cephaloleia* beetles and their native and novel host plants. (A) *Cephaloleia belti.* Native host: *Heliconia latispatha* (Heliconiaceae), Novel hosts: *H. psittacorum* (Heliconiaceae), native to the Caribbean, northern South America. *Musa velutina* (Musaceae), native to India. (B) *Cephaloleia dilaticollis.* Native host: *Renealmia alpinia.* Novel hosts: *Alpinia purpurata* (Zingiberaceae), native to the Pacific islands. *Hedychium coronarium* (Zingiberaceae), native to Eastern India. (C) *Cephaloleia placida.* Native host: *R. alpinia.* Novel hosts: *Alpinia purpurata* (Zingiberaceae), native to the Pacific islands. *Hedychium coronarium* (Zingiberaceae), native to Eastern India. (D) *Cephaloleia dorsalis.* Native host: *Costus malortieanus* (Costaceae). Novel host: *Cheilocostus speciosus* (Costaceae). Native to Eastern India.

This study was conducted from August 2005 to March 2009 at La Selva Biological Station (hereafter La Selva), a tropical rain forest in Costa Rica, Central America (10°26′N, 83°59′W). We selected four beetle species from the genus *Cephaloleia* (Chrysomelidae) with contrasting diet breadths as study species (Fig. 1). At La Selva, *Cephaloleia belti* is the species with the broadest diet breadth, feeding on 15 species from three families of Zingiberales (García-Robledo et al. 2010). The beetle *C. dilaticollis* is also a generalist, feeding on 10 species from three families of Zingiberales (García-Robledo et al. 2010). We also selected two species with specialized diets. *Cephaloleia dorsalis* feeds on four species in the family Costaceae and *C. placida* feeds on two species in the family Zingiberaceae (see a detailed description of the life cycles of these *Cephaloleia* species in García-Robledo et al. 2010).

Adults of the four *Cephaloleia* species feed on the leaf tissue of the young rolled leaves of their host plants. In contrast, larvae feed on the leaf tissue of newly expanded leaves (García-Robledo et al. 2010). At La Selva, the four beetle species have been observed feeding on exotic hosts from India, the Malay Peninsula, the Pacific Islands, and South America (Fig. 1). Invasion by exotic Zingiberales at La Selva is in an early stage. All exotic Zingiberales included in this study are common in secondary forests surrounding our study area, but very rare inside La Selva (García-Robledo and Horvitz 2011).

### Larvae acceptability and survival on native and novel host plants

Larvae of *Cephaloleia* remain on the same host plant through their development (Strong 1977). Therefore, in nature larvae of *Cephaloleia* do not experience situations where they have to select between different host plants. For this reason, we tested for differences in acceptability (estimated as differences in feeding rates in a nonchoice setup) between native and novel hosts. We also estimated differences in survival of *Cephaloleia* larvae reared on native or novel host plants. To estimate larval acceptability of native and novel hosts, we collected males and females of each *Cephaloleia* species from the native species of hosts on which larvae and adults of each beetle species were most frequently found (García-Robledo et al. 2010; Fig. 1). Mating couples were placed in separate 17 × 15 × 5 cm containers and fed ad libitum with leaf tissue from their native host plants (Number of mating couples: *N_C. belti_* = 38, *N_C. dilaticollis_* = 32, *N_C. dorsalis_* = 37, *N_C. placida_* = 42). Eggs were collected and after eclosion, larvae were randomly assigned to one of the following diets: leaf tissue from the native host or leaf tissue from one of the novel host plants (Fig. 1). Each larva was placed in an individual container and fed with two 3.5-cm diameter disks of leaf tissue. Larvae were reared at a mean temperature of 27°C and a light regime of 12 h light: 12 h (*N*_acceptability trials_ = 3728; *N*_survival trials_ = 3857; see sample sizes for each treatment in Table A1).

Differences in acceptability between native and novel hosts were estimated by measuring the area of tissue consumed by each larva 48 h after larval eclosion. Leaf area consumed was measured using a grid divided in 1 × 1 mm^2^. When there were only two host plants to compare (i.e., one native and one novel host plant), differences in area consumed between native and novel host plants were determined by using Welch two-sample *t*-tests. When there were three host plants to compare, we used one-way analyses of variance (ANOVAs).

To estimate larval survival on native and novel host plants, we fed and monitored each larva every 48 h for 259 days, by which date all of the larvae had died or pupated. Differences in larval mortality between native and novel hosts were determined by Kaplan–Meyer survival analyses.

### Adult preference and survival on native and novel host plants

Preferences and survival of adult insects can be potentially affected by their feeding experience as a larva, or by their gender (Mevi-Schutz and Erhardt 2003). For this reason, in the following experiment we include both diet as a larva and gender as factors affecting adult preference and survival. In contrast to the larvae, adults in nature do choose between host plants. Thus, this experiment both concerns choice and acceptability.

To determine the feeding preferences of adult *Cephaloleia* beetles for native or novel hosts (estimated as differences in feeding rates in a choice setup), we started with the larval stage, rearing larvae of the four species of *Cephaloleia* beetles on both the native and novel hosts (Fig. 1). Pupae were placed in individual containers. We determined the gender of each adult that emerged. For each beetle species, we simultaneously offered to each individual 3.5-cm diameter discs of leaf tissue from the native host and the novel host plants. For the specialist *C. dorsalis*, each choice trial consisted in offering leaf tissue from the native host *Costus malortieanus* and the novel host *Cheilocostus speciosus.* For the other beetle species, we simultaneously offered leaf tissue from their native host and two novel host plants (Fig. 1). Individual beetles were only used in one trial. After 12 h, we measured the leaf area consumed using a grid divided in 1 × 1 mm^2^. To test for differences in leaf tissue consumed on native and novel host plants, for each beetle species we performed a two-way ANOVA where each adult feeding on native and novel hosts were treated as a block. Diet as a larva and gender were included as fixed factors (*N*_adults_ = 823; see sample sizes for each treatment in Table A2).

To determine differences in survival of adults feeding on native or novel hosts, we obtained adults of each beetle species from larvae reared on native or novel hosts (Fig. 1). The gender of each adult was recorded before being placed in individual containers. Adults were fed ad libitum with leaf tissue of either their native or their novel host plant. Time to death was recorded by monitoring each beetle every 48 h for 429 days, by which date all of the beetles had died. We explored differences in adult survival among diets with a fully crossed ANOVA design that included diet as larva, diet as adult, and gender as fixed factors. The response variable was the square root transformed time to death (*N* = 551; see sample sizes for each analysis level and treatment in Table A3).

### Oviposition preferences on native and novel host plants

To determine the oviposition preferences of female *Cephaloleia* on native and exotic Zingiberales, we collected females from their native hosts (Fig. 1). Females were brought to the laboratory, and mated with a male. Because exotic Zingiberales are extremely rare inside the study site, most likely females did not have previous feeding or oviposition experience on the novel hosts (García-Robledo and Horvitz 2011). Each mating couple was placed in an independent arena (40 × 20 × 50 cm). Each arena contained two trays (17 × 15 × 5 cm). Each tray contained leaf tissue of either the native or a novel host plant (Fig. 1). Although females feed on young leaves, they use expanded leaves as the oviposition substrate. Therefore, each tray was filled with 200 cm^2^ of leaf tissue from fully expanded leaves. We also included in each tray 100 cm^2^ of leaf tissue from an unexpanded leaf. To estimate oviposition preferences, we recorded after 72 h the number of eggs laid on leaves of native or novel host plants. Differences in oviposition were explored using paired *t*-tests (see sample size in Table 1).

**Table 1:** Paired *t*-tests exploring differences in the number of eggs laid on native and novel hosts after 72 h. Significant results (*P* < 0.05 are highlighted in bold). See comparisons between means in Figure 4

Beetle species	Host plant^1^ combination	*t*	*N*^2^	*n*^2^	*P*
*C. belti* (generalist)	HL _(Native)_–HP _(Novel)_	2.3527	40	26	**0.02**
	HL _(Native)_–MV _(Novel)_	3.5147	21	10	**<0.01**
*C. dilaticollis* (generalist)	RA _(Native)_–AP _(Novel)_	0	22	15	1
	RA _(Native)_–HC _(Novel)_	0.6828	34	23	0.5
*C. dorsalis* (specialist)	CM _(Native)_–CS _(Novel)_	–0.474	31	17	0.64
*C. placida* (specialist)	RA _(Native)_–AP _(Novel)_	4.2495	33	22	**<0.001**
	RA _(Native)_–HC _(Novel)_	1.0059	33	22	0.32

**Figure 2:**
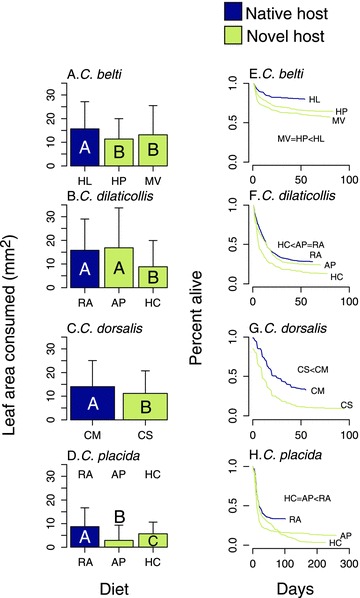
Larval acceptability (mean ± SD) and survival of *Cephaloleia* beetles on native and novel host plants. (A and E) Acceptability and survival of the generalist beetle *C. belti* on the native host *H. latispatha* (HL) and the novel hosts *H. psittacorum* (HP) and *M. velutina* (MV). (B and F) Acceptability and survival of the generalist beetle *C. dilaticollis* on the native host *R. alpinia* (RA) and the novel hosts *A. purpurata* (AP) and *H. coronarium* (HC). (C and G) Acceptability and survival of the specialist beetle *C. dorsalis* on the native host *C. malortieanus* (CM) and the novel host *Ch. speciosus* (CS). (D and H) Acceptability and survival of the specialist beetle *C. placida* on the native host *R. alpinia* (RA) and the novel hosts *A. purpurata* (AP) and *H. coronarium* (HC). Letters on the columns group similar categories. See sample sizes in Table A1.

1 Host plant abbreviations as in Figure 2.

2 Sample size: *N* is the number of couples selected for each experiment; *n* is the total number of females that oviposited.

## Results

### Larval acceptability and performances on native and novel hosts

In general, larvae consumed more leaf tissue of native hosts than of novel hosts and larval survival was higher on the native than the novel host plants (see summary of results in Table 2). Newborn larvae of the generalist beetle *C. belti* consumed 16–27% more leaf tissue from the native host, *Heliconia latispatha* than from the novel hosts *H. psittacorum* and *Musa velutina* (*F*_2_ = 13.55, *P* < 0.0001, Fig. 2A). Larval survival of *C. belti* was 16–23% higher on the native than on the novel hosts (χ^2^ = 44.82, df = 2, *P* < 0.001, Fig. 2E).

**Table 2:** Summary of results for experiments comparing life-history traits and demographic parameters associated with oviposition preferences on native and novel hosts. Positive associations between oviposition preferences and life-history traits/vital rates are highlighted in bold

Species	Original host	Novel host	Larval acceptability^1^	Larval survival^1^	Adult preferences^1^	Adult longevity^1^	Female fecundity^2^	Instantaneous population growth rate (*r*)^2^	Oviposition preference^1^
*C. belti* (generalist)									
	*H. latispatha* (HL)								
		*H. psittacorum* (HP)	**HL > HP**	**HL > HP**	HL ≤ HP	HL < HP	**HL ≥ HP**	**HL > HP**	**HL > HP**
		*M. velutina* (MV)	**HL > MV**	**HL > MV**	HL < MV	HL = MV	**HL > MV**	**HL > MV**	**HL > MV**
*C. dilaticollis* (generalist)									
	*R. alpinia* (RA)								
		*A. purpurata* (AP)	**RA = AP**	**RA = AP**	RA < AP	RA < AP	**RA = AP**	**RA = AP**	**RA = AP**
		*H. coronarium* (HC)	RA > HC	RA > HC	**RA = HC**	RA > HC	–3	RA > HC	**RA = HC**
*C. dorsalis* (specialist)									
	*C. malortieanus* (CM)								
		*Ch. speciosus* (CS)	CM > CS	CM > CS	CM < CS	**CM = CS**	**CM = CS**	CM > CS	**CM = CS**
*C. placida* (specialist)									
	*R. alpinia* (RA)								
		*A. purpurata* (AP)	**RA > AP**	**RA > AP**	**RA ≥ AP**	RA = AP	**RA > AP**	**RA > AP**	**RA > AP**
		*H. coronarium* (HC)	RA > HC	RA > HC	RA > HC	RA > HC	–3	RA > HC	**RA = HC**

1 Results summarized from this paper.

2 Results summarized from García-Robledo and Horvitz 2011.

3 Not enough females available to perform fecundity comparisons (García-Robledo and Horvitz 2011).

Larvae of the generalist beetle *C. dilaticollis* consumed two times more leaf tissue of its native host *Renealmia alpinia* and the novel host *Alpinia purpurata* than of the novel host *Hedychium coronarium* (*F*_2_ = 21.41, *P* < 0.0001, Fig. 2B). Larval survival of *C. dilaticollis* was 11–15% higher on the native hosts *R. alpinia* and on the novel host *A. purpurata* than on the novel host *H. coronarium* (χ^2^ = 26.02, df = 2, *P* < 0.001, Fig. 2F).

The feeding rates of larvae reared on the native host *C. malortieanus* were 21% higher in the native host *C. malortieanus* than the novel host plant *Ch. speciosus* (Welch two-sample *t*-test, df = 646.9, *t* = 3.54, *P* = 0.0004, Fig. 2C). Larval survival of *C. dorsalis* was 10% higher on the native host *C. malortieanus* than on the novel host *Ch. speciosus* (*Z* = 3.44, df = 1, *P* < 0.001, Fig. 2G).

Feeding rates of larvae of the specialist beetle *C. placida* reared on the native host *R. alpinia* were 35–67 % higher than on the novel hosts *A. purpurata* and *H. coronarium* (*F*_2_ = 46.39, *P* < 0.0001, Fig. 2D). Larval survival of *C. placida* was higher on the native host *R. alpinia* than on the novel host *A. purpurata* and *H. coronarium* (χ^2^ = 28.78, df = 2, *P* < 0.001, Fig. 2H).

### Adult preference and performances on native and novel hosts

#### Effects of larval diet on adult preference and longevity

For the four beetle species, host selection and survival of adults reared on native or novel hosts were not affected by their feeding experience as a larva. For the generalist species *C. belti*, we were able to obtain adults from larvae reared on the native host *H. latispatha,* as well as from larvae reared on the novel hosts *H. psittacorum* and *M. velutina.* In *C. belti* adult preferences (*F*_2, 467_ = 0.99, *P* = 0.37) and longevity (*F*_2_ = 1.56, *P* = 0.21) were not affected by the diet experienced during larval stages.

For the generalist species *C. dilaticollis*, mortality of larvae reared on one of the two novel hosts, *H. coronarium*, was exceptionally high (mortality = 87.4%; see sample sizes for each factor in Table A1). For this treatment, very few adults were available. For this reason, we restricted our studies of the effect of larval diet on adult preference and longevity to larvae reared on the native host *R. alpinia* and the novel host *A. purpurata*, for which we obtained sufficient adults. Adult host choice preferences (*F*_1, 185_ = 0.56, *P* = 0.45) and longevity (*F*_1_ = 0.0.38, *P* = 0.54) were not affected by the diet of the beetles during the larval stage.

For the specialist species *C. dorsalis*, we were able to obtain adults from larvae reared on both the native host *C. malortieanus* and the novel host *Ch. speciosus.* Host plant choice (*F*_1, 95_ = 3.74, *P* = 0.06) and longevity (*F*_1_ = 0.18, *P* = 0.67) of adult beetles on native and novel hosts are not affected for the diet as larvae in this beetle species.

For the specialist beetle species *C. placida*, we obtained sufficient adults only from larvae raised on the native host *R. alpinia.* Mortality of larvae reared on both novel hosts *A. purpurata* and *H. coronarium* was exceptionally high (mortality in *A. purpurata* = 85.9%, mortality in *H. coronarium* = 96.7%; see sample sizes in Table A1). Therefore, for this species we were not able to analyze the effects of larval diet on adult preference and longevity.

#### Effect of gender on adult preference and longevity

For all species, males and females made the same choices in host preference trials. However, for some species, longevity was different among genders. Males of the specialist species lived longer than females. Males and females of the generalist species *C. belti* and *C. dilaticollis* displayed similar host preferences (*C. belti*: *F*_1, 467_ = 0.85, *P* = 0.36, *C. dilaticollis: F*_1, 185_ = 0.068, *P* = 0.79). Generalist males and females lived as long on native as they did on novel hosts (*C. belti*: *F*_1_ = 0.35, *P* = 0.55, *C. dilaticollis: F*_1_ = 0.58, *P* = 0.44).

Males and females of the specialist species *C. dorsalis* and *C. placida* did not differ in host choice preferences (*C. dorsalis*: *F*_1, 95_ = 0.45, *P* = 0.50, *C. placida: F*_1, 36_ = 0.17, *P* = 0.68). However, males of these species lived longer than females (*C. dorsalis*: *F*_1_ = 10.28, *P* = 0.002, mean ± SD_females_ = 162 ± 83.72 days, mean ± SD_males_ = 223.58 ± 97.71 days; *C. placida: F*_1_ = 4.05, *P* = 0.04, mean ± SD_females_ = 78.76 ± 64.26 d., mean ± SD_males_ = 98.79 ± 114.31 days; see sample size in Table A3).

#### Adult preference and longevity on native and novel host plants

**Figure 3:**
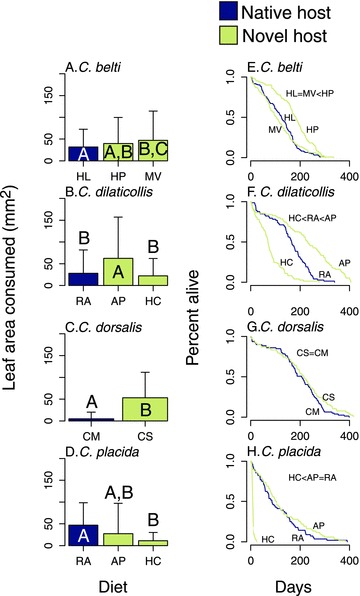
Adult preference (mean ± SD) and survival of *Cephaloleia* beetles in native and novel host plants. (A and E) *Cephaloleia belti* (generalist), (B and F) *Cephaloleia dilaticollis* (generalist), (C and G) *Cephaloleia dorsalis* (specialist), (D and H) *Cephaloleia placida* (specialist). Letters on the columns group similar categories. See sample sizes in Tables A2 and A3. Host plant abbreviations as in Figure 2.

In general, adults display equivalent preferences and longevity on both native and novel hosts, or may even prefer and live longer on the novel than on the native host plants (see summary of results in Table 2). Adults of the generalist species *C. belti* preferred to feed on their novel hosts (*F*_2, 904_ = 9.12, *P* = 0.0001, Fig. 3A). Adults of *C. belti* lived longer on the novel host *H. psittacorum* than on the native host *H. latispatha* and the novel host *M. velutina* (*F*_2_ = 20.53, *P* < 0.0001, Fig. 3E).

Adults of the generalist beetle *C. dilaticollis* preferred to feed on the novel host *A. purpurata* over the native host *R. alpinia* and the novel host *H. coronarium* (*F*_2, 185_ = 12.38, *P* < 0.0001, Fig. 3B). *Cephaloleia dilaticollis* lived longer on the novel host *A. purpurata* than on the native host *R. alpinia.* Adults of *C. dilaticollis* lived shorter when feeding on the novel host *H. coronarium* (*F*_2_ = 28.72, *P* < 0.0001, Fig. 3F).

Adults of the specialist beetle *C. dorsalis* preferred to feed on the novel host *Ch. speciosus* over the native host *C. malortieanus* (*F*_1, 64_ = 111.68, *P* < 0.0001, Fig. 3C). Adult survival was equivalent in both host plants (*F*_1_ = 0.08, *P* = 0.77, Fig. 3G).

Adults of the specialist beetle species *C. placida* preferred to feed on its native host plant *R. alpinia* and the novel host *A. purpurata* over the novel host *H. coronarium* (*F*_2, 45_ = 7.00, *P* < 0.002, Fig. 3D). Adult longevity was higher on the native host *R. alpinia* and the novel host *A. purpurata* than on the novel host *H. coronarium* (*F*_2_ = 57.31, *P* < 0.0001, Fig. 3H).

### Oviposition preferences on native and novel hosts

**Figure 4:**
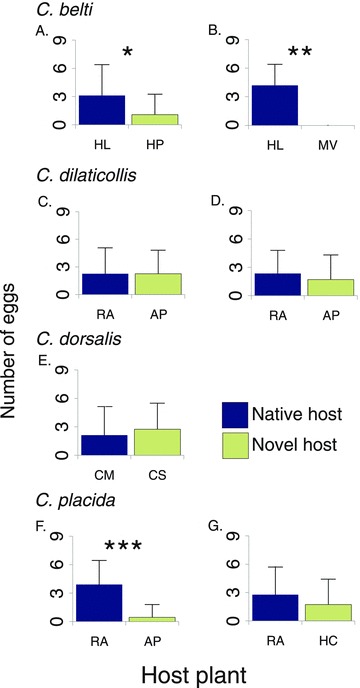
Number of eggs oviposited on native and novel host plants by female *Cephaloleia* during oviposition preference experiments (mean ± SD). (A and B) *Cephaloleia belti* (generalist), (C and D) *Cephaloleia dilaticollis* (generalist), (E) *Cephaloleia dorsalis* (specialist), (F and G) *Cephaloleia placida* (specialist). See sample size in Table 1. *P < 0.05; **P < 0.01;***P < 0.001. Host plant abbreviations as in Figure 2.

In general, when females showed oviposition preferences for a host plant, they oviposited on their native hosts (Fig. 4; see summary of results in Table 2). The generalist beetle *C. belti* prefers to oviposit on its native host *H. latispatha* rather than on the novel hosts *H. psittacorum* and *M. velutina* (Table 1; Fig. 4A and B). The generalist beetle *C. dilaticollis* laid equivalent numbers of eggs on both native and novel hosts (Table 1; Fig. 4C and D).

The specialist beetle *C. dorsalis* laid equivalent number of eggs on native and novel host plants (Table 1; Fig. 4E). The specialist beetle *C. placida* laid more eggs on the native host *R. alpinia* than on the novel host *A. purpurata* (Table 1; Fig. 4F). The number of eggs laid by *C. placida* on the native host *R. alpinia* and the novel host *H. coronarium* were equivalent (Table 1; Fig. 4G).

## Discussion

This study explores the behavioral and physiological responses of generalist and specialist *Cephaloleia* beetles expanding their diets to novel hosts. Our results give an insight into the processes assembling these novel plant–herbivore interactions. *Cephaloleia* beetle preferences and physiology are in some degree preadapted to novel plants. It is remarkable that although *Cephaloleia* beetles are neotropical, this group of herbivores is preadapted to host plants that evolved isolated in the paleotropics for the last 40–60 million years (Wilf et al. 2000; García-Robledo and Staines 2008). This suggests that *Cephaloleia* beetles can colonize novel hosts without substantial evolutionary change after encountering a potential host plant.

Although *Cephaloleia* preferences and physiology are in some degree preadapted to novel hosts, expansions of *Cephaloleia* beetles are conservative. Novel associations were restricted to plants from the same plant families of the native hosts. Specialization to a particular plant family is a common feature among *Cephaloleia* species at La Selva Biological Station in Costa Rica and the Soberania National Park, a tropical rain forest in Panama, Central America (García-Robledo and Horvitz 2009; García-Robledo 2010; García-Robledo et al. 2010; Meskens et al. 2011). Initial colonization of novel hosts at higher taxonomic ranks by *Cephaloleia* beetles is apparently a rare event in ecological time. However, evidence from molecular phylogenetics studies suggests that *Cephaloleia* lineages shifted their diets to different plant families several times during their diversification (McKenna and Farrell 2005). Previous studies show that after plant introduction, local herbivores can rapidly colonize novel hosts. Some examples are plant crops and exotic plants (Strong 1979; Maron and Vilà 2001). In most cases, herbivore diet expansions are limited to plants closely related to their original hosts. Such patterns are evident at both ecological and evolutionary scales (Strong 1979; Maron and Vilà 2001; Price 2008).

In this research, we explored the initial feeding preferences and performance of insect herbivores during the early colonization of novel hosts. Current theory predicts that initial preferences and performance of insect herbivores should be: (1) higher on native than on novel hosts as a result of local adaptation to native hosts (Chew 1977; Strauss et al. 2006) or (2) initial choices are maladaptive, as insect herbivores did not have enough time to adapt to the novel host plants (Schlaepfer et al. 2002; Keeler and Chew 2008; Harvey et al. 2010).

Our results suggest a more complex scenario. For generalist and specialist *Cephaloleia* beetles, feeding preferences are usually associated with hosts that increase insect survival. However, life stages display different responses when feeding on native or novel hosts. Larval preferences and survival are usually higher on native than on novel hosts. Adults display the opposite response, in some cases having an increase in longevity when feeding on novel hosts.

Larval and adult behavioral and physiological responses to novel hosts represent antagonistic processes that may exert opposite effects on insect herbivore's diet breadth. Larval preference for native hosts should promote local adaptation and generate narrow diet breadths. Adult preference and high performance on novel hosts will promote diet generalization. This suggests that the evolution of diet breadths can be the result of selection acting on conflicting life-history trade-offs in performance between larval and adult life stages.

Previous studies focused on the relationships between oviposition preferences and offspring performance (Craig and Itami 2008). Unfortunately very few studies estimated the effect of host consumption on adult longevity (Gripenberg et al. 2010). A previous study explored the trade-offs between parent and offspring performance and oviposition preferences in the leafmining fly *C. nigra* (Scheirs et al. 2000). In this insect species, females prefer to oviposit on hosts that maximize adult, not offspring survival. This study challenged current approaches used to understand relationships between oviposition preferences and life-history traits of insects. If we aim to understand the processes selecting for oviposition preferences, we must research the associations between oviposition choices and insect fitness, not only one fitness component such as offspring survival (Mayhew 2001).

Differences between larval and adult preferences and performance on novel hosts are generating initial parent–offspring conflicts during *Cephaloleia* diet expansions. One behavior that could ameliorate such conflicts is larval migration to more suitable host plants (Craig and Itami 2008; Gripenberg et al. 2010). This is not possible for larvae of *Cephaloleia* beetles, which rarely migrate from the host plants selected by their mothers (Strong 1982a, b). Therefore, the outcome of these initial parent–offspring conflicts will depend on the decisions of females to oviposit on either the native or the novel host (Mayhew 2001).

In this study, we found no evidence for female oviposition preferences predicted by the “optimal bad motherhood” principle. When female *Cephaloleia* displayed any oviposition preference, they selected native host, as predicted by the “mother knows best” principle. This suggests that preadapted feeding preferences and physiology of adults facilitate the exploration and colonization of novel hosts, but oviposition preferences are conservative.

In a meta-analysis that estimated the effects of several plant and insect life-history traits on oviposition preferences–offspring performance associations, the only factor associated with oviposition preferences was diet breadth (Gripenberg et al. 2010). In this meta-analysis, specialist herbivores trend to have stronger oviposition preference–offspring performance relationships than generalist species. The authors suggest that specialist females oviposit on hosts that increase offspring performance as a result of higher local adaptation to their hosts than generalist herbivores (Gripenberg et al. 2010). Another possibility suggested by this and other studies is that generalists display neural system constrains (Craig and Itami 2008). Neural limitation theory assumes that insects are able to recognize a limited number of host plants and oviposition mistakes increase in species with a higher number of host plants (Bernays 2001).

If local adaptation or neural constraints differentially affect generalist and specialist oviposition choices, a prediction for insect herbivores colonizing novel hosts is that specialists will perform better oviposition decisions than generalists. Based on our results, this is not the case for generalist and specialist *Cephaloleia* beetles.

One of the goals of this research was to determine associations between life-history traits and oviposition preferences during early stages of insect diet expansions. As shown in our summary of results (Table 2), for more than half of the novel plant–herbivore interactions included in this study, ovi-position preferences are associated with larval survival. Ovi-position preference is associated with adult longevity in only one plant–herbivore interaction (Table 2). Our conclusion is that oviposition preferences of *Cephaloleia* beetles are more frequently associated with larval than with adult performance during the colonization of novel hosts.

Larval survival is not the only life-history trait associated with oviposition preferences. In a previous study, we recorded differences in fecundities of female *Cephaloleia* reared on native or novel hosts (García-Robledo and Horvitz 2011; see summary of results in Table 2). When contrasting our results on oviposition preferences with fecundity estimates on native and novel hosts, we found that females prefer to oviposit on plants that also increase their fecundities (Table 2).

Larval and adult survival are two important life-history traits. However, they are only two components determining fitness on native and novel hosts. A more insightful approach would be to determine if female oviposition preferences are associated with the full pattern of vital rates that determine insect fitness on a given host plant (Craig and Itami 2008). One parameter that estimates individual's fitness in a given environment is the instantaneous population growth rate (McGraw and Caswell 1996). In a previous study, we estimated the instantaneous population growth rates for *Cephaloleia* beetles on native and novel hosts (García-Robledo and Horvitz 2011; see summary of results in Table 2). For more than half of the novel interactions, female oviposition preferences are associated with the instantaneous population growth rates attained in a particular host plant (Table 2).

This study explores during early host colonizations if oviposition host choices are maladaptive, conservative, or follow the oviposition hierarchies predicted by the “optimal bad motherhood” or the “mother knows best” principles. Our main conclusion is that oviposition preferences are in most cases associated with hosts that increase larval survival, female fecundity, and population growth. This suggests that during the colonization of novel hosts, female *Cephaloleia* are not optimal bad mothers, in most cases *Cephaloleia* ovi-position preferences follow the expectations of the “mothers knows best” principle.

## Appendix

**Table A1:** Sample sizes for experiments testing differences in acceptability and survival in larvae of *Cephaloleia* reared on native and novel host plants

		Number of larvae	
		
Herbivore species	Diet	Acceptability trials	Survival trials
*Cephaloleia belti* (38^1^)			
	*H. latispatha* (Native)	334	348
	*H. psittacorum* (Novel)	374	389
	*M. velutina* (Novel)	391	397
*Cephaloleia dilaticollis* (32^1^)			
	*R. alpinia* (Native)	545	574
	*A. purpurata* (Novel)	526	541
	*H. coronarium* Novel)	184	213
*Cephaloleia dorsalis* (37^1^)			
	*C. malortieanus* (Native)	337	340
	*Ch. speciosus* (Novel)	316	323
*Cephaloleia placida* (42^1^)			
	*R. alpinia* (Native)	268	272
	*A. purpurata* (Novel)	214	220
	*H. coronarium* (Novel)	239	240

1 Number of females collected in the field from which larvae were obtained for acceptability and survival trials.

**Table A2:** Sample sizes for experiments testing differences in feeding preferences between native and novel hosts for male and female adults of *Cephaloleia*. Adults were obtained from larvae reared on either native or novel host plants

		Number of adults	
		
Herbivore species	Diet as a larva	Females	Males
*Cephaloleia belti*			
	*H. latispatha* (Native)	96	103
	*H. psittacorum* (Novel)	82	81
	*M. velutina* (Novel)	77	68
*Cephaloleia dilaticollis*			
	*R. alpinia* (Native)	32	25
	*A. purpurata* (Novel)	26	35
	*H. coronarium* (Novel)	–1	–1
*Cephaloleia dorsalis*			
	*C. malortieanus* (Native)	39	42
	*Ch. speciosus* (Novel)	20	30
*Cephaloleia placida*			
	*R. alpinia* (Native)	32	35
	*A. purpurata* (Novel)	–1	–1
	*H. coronarium* (Novel)	–1	–1

1 Trials not performed because larval mortality was high, precluding obtaining the required adults for preference trials.

**Table A3:** Sample sizes for experiments testing differences in longevity in female and male adults of *Cephaloleia* feeding on native or novel hosts. Adults were obtained from larvae reared on either native or novel host plants

			Number of adults	
			
Beetle species	Diet as a larva	Diet as adult	Females	Males
*Cephaloleia belti*				
	*H. latispatha* (Native)			
		*H. latispatha* (Native)	23	22
		*H. psittacorum* (Novel)	18	22
		*M. velutina* (Novel)	20	22
	*H. psittacorum* (Novel)			
		*H. latispatha* (Native)	17	21
		*H. psittacorum* (Novel)	17	18
		*M. velutina* (Novel)	18	22
	*M. velutina* (Novel)			
		*H. latispatha* (Native)	18	18
		*H. psittacorum* (Novel)	17	18
		*M. velutina* (Novel)	20	19
*Cephaloleia dilaticollis*				
	*R. alpinia* (Native)			
		*R. alpinia* (Native)	20	22
		*A. purpurata* (Novel)	20	14
		*H. coronarium* (Novel)	20	21
	*A. purpurata* (Novel)			
		*R. alpinia* (Native)	13	14
		*A. purpurata* (Novel)	14	14
		*H. coronarium* (Novel)	13	16
	*H. coronarium* (Novel)			
		*R. alpinia* (Native)	–1	–1
		*A. purpurata* (Novel)	–1	–1
		*H. coronarium* (Novel)	–1	–1
*Cephaloleia dorsalis*				
	*C. malortieanus* (Native)			
		*C. malortieanus* (Native)	17	21
		*Ch. speciosus* (Novel)	16	20
	*Ch. speciosus* (Novel)			
		*C. malortieanus* (Native)	24	14
		*Ch. speciosus* (Novel)	16	20
*Cephaloleia placida*				
	*R. alpinia* (Native)			
		*R. alpinia* (Native)	34	17
		*A. purpurata* (Novel)	35	19
		*H. coronarium* (Novel)	19	21
	*A. purpurata* (Novel)			
		*R. alpinia* (Native)	–1	–1
		*A. purpurata* (Novel)	–1	–1
		*H. coronarium* (Novel)	–1	–1
	*H. coronarium* (Novel)			
		*R. alpinia* (Native)	–1	–1
		*A. purpurata* (Novel)	–1	–1
		*H. coronarium* (Novel)	–1	–1

1 Trials not performed because larval mortality was high, precluding obtaining the required adults for longevity trials.
